# Association of SGLT-2 inhibitors with bacterial urinary tract infection in type 2 diabetes

**DOI:** 10.1186/s12902-023-01464-6

**Published:** 2023-10-03

**Authors:** Mustafa Tanrıverdi, Mehmet Baştemir, Hadiye Demirbakan, Alperen Ünalan, Merve Türkmen, Gülşen Özkan Tanrıverdi

**Affiliations:** 1 Department of Infectious Diseases, SANKO University Faculty of Medicine, Gazimuhtar Paşa Bulvar? No:36 - 27090 Şehitkamil, Gaziantep, Turkey; 2 Department of Medical Microbiology, SANKO University Faculty of Medicine, Gaziantep, Turkey; 3 Department of Internal Medicine, SANKO University Faculty of Medicine, Gaziantep, Turkey; 4 Department of Anesthesiology and Reanimation, 25 Aralık State Hospital, Gaziantep, Turkey

**Keywords:** SGLT-2, Inhibitor, Diabetes, Type 2, Infection, Urinary

## Abstract

**Objective:**

We aimed to investigate the factors associated with UTI in patients with T2D whether being treated with SGLT-2i or not.

**Methods:**

Adult patients with T2D, whose urine culture results were available, were analyzed retrospectively. Urine culture was obtained from mid-flow urine. Antibacterial treatment was given to the patients with UTI, which was defined by positive urine cultures and/or clinical findings. We grouped the patients as follows: Group A, those treated with SGLT-2i; and Group B, those not treated with SGLT-2i.

**Results:**

A total of 101 patients were included. Median age was 56 (45–67), 56.4% (n = 57) of the patients were female. Urine culture was positive in 54.9% (n = 28) and 16% (n = 8) of Group A (n = 51) and Group B (n = 50), respectively. Of those for whom urine culture was positive, Escherichia coli was isolated in 83.3% (n = 30), and both Escherichia coli and Klebsiella pneumoniae (K.pneumoniae) were isolated in 16.7% (n = 6). Klebsiella pneumoniae was isolated only from Group A. The need for and duration of hospitalization were higher in Group A (p < 0.001). UTI was detected in 60 patients. ROC analysis showed that a HbA1c of > 5.8% was associated with UTI with good accuracy (AUC: 0.835, p < 0.001). In multiple logistic regression analysis, SGLT-2i use and glucosuria were positive predictors for UTI (p = 0.004, Odds Ratio: 1984.013; and p = 0.028, and Odds Ratio: 12.480, respectively).

**Conclusion:**

Besides the association of HbA1c and BMI with UTI, SGLT-2i use and glucosuria predicted UTI. Urine culture is important with respect to the choice of antibacterial treatment, especially in those patients under SGLT-2i treatment. The effect of SGLT-2i on the development of UTI is independent of baseline BMI score or HbA1c.

## Introduction

Sodium glucose co-transporter 2 inhibitors (SGLT-2i) have been frequently used in the management of type 2 diabetes (T2D) for a decade [1]. They exert their effect via inhibition of sodium glucose transporter 2 (SGLT-2) in the proximal tubule of the kidney, independent of insulin, increasing urinary glucose excretion [2]. SGLT-2i were shown to be associated with an increased risk of genital mycotic infections, especially in those with a previous history of genital infections [3–5]. They also increased the risk of uncomplicated urinary tract infection (UTI), urosepsis or pyelonephritis [1, 3]. The findings indicating the association of UTI with SGLT-2i were not as consistent as that concerning the association of genital infections with SLGT-2i use [3]. Dapagliflozin and empagliflozin was shown to increase the risk of UTI in many studies including a DURATION-8 study [1, 3, 6, 7]. Some studies showed that SGLT-2i use was not associated with an increased risk of UTI [4, 5, 8–15]. In a recent meta-analysis of a considerable number of randomized trials analyzing SGLT-2i use, the risk of UTI was higher where higher doses of dapagliflozin were administered [16]. In one study, UTI incidence was shown to be 4–9%, and severe infection 0.4% after SGLT-2i use [17–20].

Although the mechanism underlying UTI associated with SGLT-2i has not been clearly defined, glucosuria, effect of SGLT-2i, may provide a microorganism-favoring environment [1, 21]. However, osmotic diuresis, by maintaining urinary flow, may counteract the microorganism-favoring milieu [22]. Adequate fluid intake was an in consistent factor, which might be associated with UTI [23].

The factors, which have a possible role in the development of UTI after SGLT-2i use in T2D, have been less studied. We aimed to investigate the clinical and laboratory factors associated with UTI, which occurred after SGLT-2i use in those patients with T2D.

## Materials and methods

### Study design

This retrospective study was conducted in SANKO University hospital, and approved by the local Ethics Committee of SANKO University (Date: 2021/11 Decision No: 02). Written informed consent was obtained from all of the participants.

Adult patients diagnosed and followed-up with T2D in SANKO University hospital between January 2020 and December 2020, and whose urine culture results were available, were evaluated and analyzed retrospectively. The diagnosis and management of T2D was based on ADA guidelines [24, 25]. Those patients with any contraindications to treatment with SGLT-2i at the baseline were not given SGLT-2i [24, 25]. All patients underwent clinical and laboratory evaluation. Those patients with other types of diabetes mellitus, younger than 18 years of age, or those for whom data were missing were not included in the study. Benign prostate hypertrophy was known to be one of the causes of urinary tract infection in men. Therefore, male patients with a history of prostate hypertrophy were excluded. Those patients with genital infections or for whom a fungal agent was isolated before the study were excluded, because it might lead bias.

### Data collection

Demographic parameters (age, sex, and body mass index [BMI]), smoking status, clinical parameters (symptoms [fever, dysuria, pollakuria, urgency, and nausea-vomiting], history of hypertension, need for hospitalization, duration of hospitalization [days]), and laboratory findings (complete blood count, CRP, fasting blood glucose [FBG], HbA1c, and urinalysis [pyuria, hematuria, bacteriuria, glucosuria, proteinuria, and nitrit positivity]) were recorded by using electronic and written patient files. Urine culture was obtained from mid-flow urine for each participant. Positivity of urine culture, species of bacteria isolated from urine culture, and antibacterial treatment were also recorded and analyzed. Urine culture was defined as positive if at least 10^5^ colonies/mL was present.

Antibacterial treatment was given for 7–10 days to those patients in whom a UTI was detected. UTI was defined based both on the result of positive urine cultures and clinical signs and symptoms. To reveal a negative urine culture, a control urine culture was performed after antibacterial treatment in hospitalized patients.

Hospitalization was needed in unstable patients, those requiring intravenous antibacterial treatment, or with complicated UTIs. Pyelonephritis or urosepsis was not detected in any patients.

A complete blood count was studied with an automated analyzer. Urinalysis and FBG were measured by a glucose oxidase method with the Olympus AU-2700 analyzer. HbA1c was measured as NGSP (National Glycohemoglobin Standardization Program) units by the HPLC method (high purification liquid chromatography).

### Patient groups

Patients were grouped based on SGLT-2i treatment: Group A, those treated with SGLT-2i; and Group B, those not treated with SGLT-2i. The participants in Groups A and B were given other antidiabetic medications according to the ADA guidelines, with the exception of insulin [25]. In those patients under SGLT-2i treatment, SGLT-2i had been used for at least 1 month. Empagliflozin or dapagliflozin at a dose of 10 mg/day was given in Group A. During the follow-up, mortality was also observed and analyzed. We compared demographic, clinical and laboratory parameters between the groups.

### Statistical analysis

Data obtained in the study were analysed statistically using SPSS 26.0 (IBM Corporation, Armonk, New York, United States). The conformity of the data to normal distribution was evaluated using the Shapiro-Wilk francia test. When comparing two independent groups of quantitative data according to each other, we used the nonparametric Mann-Whitney U test with Monte Carlo results. When comparing categorical variables with each other, the Pearson Chi-square, Fisher’s exact and Fisher-Freeman-Holton tests with the Monte Carlo simulation technique were used. Comparison of column ratios with each other was expressed by Benjamini-Hochberg corrected p-values. ROC curve analysis was performed for quantitative parameters significant for urinary tract infection. To detect a cause-effect relationship for urinary tract infection, logistic regression analysis was performed with the Enter method. Quantitative variables were stated as mean (standard deviation), and median (minimum-maximum) values, and categorical variables as number (n) and percentage (%) in the tables. Variables were evaluated at a 95% confidence level, and a value of p < 0.05 was accepted as statistically significant.

## Results

A total of 101 patients with T2D were included in the study. Group A consisted of 51 patients, and Group B 50 patients. Dapagliflozin was prescribed for 31 patients, and Empagliflozin for 20 patients in Group A. The median age was 56 (45–67), 56.4% (n = 57) of the patients were female. Hypertension was found in 56.4% (n = 57) of the patients. The BMI score was higher in Group A (p < 0.001). Fever, dysuria, pollakiuria, urgency, nausea-vomiting, pyuria, bacteriuria, and glucosuria were more frequent in Group A. Hematuria was not detected in any patients. Nitrite in urinalysis was positive in 27.5% (n = 14) of the patients in Group A, and 2% (n = 1) in Group B (p < 0.001).

Urine culture was positive in 35.6% (n = 36), 54.9% (n = 28), and 16% (n = 8) of the entire group, Group A and Group B, respectively. Among those for whom urine culture was positive, Escherichia coli was isolated in 83.3% (n = 30), and both Escherichia coli and Klebsiella pneumoniae were isolated in the same urine culture in 16.7% (n = 6) of the patients. Klebsiella pneumoniae was isolated only from Group A. 60 patients were treated with antibacterial medications. Antibacterial medications were similar in 2 groups. The need for hospitalization and the duration of hospitalization were higher in Group A (p < 0.001 and p < 0.001, respectively) (Table 1).

**Table 1:** Demographic, clinical and laboratory features of the patients

	Total (n = 101)	Group A SGLT-2i (+) (n = 51)	Group B SGLT-2i (-) (n = 50)	P
	n (%) or median (min-max)	n (%) or median (min-max)	n (%) or median (min-max)	
Sex (female)	57 (56.4)	28 (54.9)	29 (58)	0.842 ^c^
Age (year)	56 (45–67)	56 (45–66)	56.5 (45–67)	0.370 ^u^
BMI (kg/m^2^)	32 (24–41)	32 (25–41)	28.5 (24–34)	**0.001** ^**u**^
Smoking (present)	40 (39.6)	15 (29.4)	25 (50)	**0.043** ^**c**^
Hypertension (present)	57 (56.4)	29 (56.9)	28 (56)	0.999 ^c^
Fever (present)	27 (26.7)	18 (35.3)	9 (18)	**0.071** ^**c**^
Dysuria (present)	60 (59.4)	51 (100)	9 (18)	**< 0.001** ^**c**^
Pollakiuria (present)	60 (59.4)	51 (100)	9 (18)	**< 0.001** ^**c**^
Urgency (present)	54 (53.5)	45 (88.2)	9 (18)	**< 0.001** ^**c**^
Nausea-Vomiting (present)	27 (26.7)	18 (35.3)	9 (18)	**0.071** ^**c**^
HbA1c (%)	6.8 (5.6–11)	7.8 (6.1–11)	5.8 (5.6–11)	**< 0.001** ^**u**^
FBG (mg/dL)	156 (112–234)	156 (112–234)	145 (112–234)	0.160 ^u^
Leukocyte (/mm^3^)	12,540 (2300–23,800)	15,600 (10,800–23,800)	7700 (2300–21,000)	**< 0.001** ^**u**^
CRP (mg/dL)	45 (1-145)	89 (37–145)	5 (1-123)	**< 0.001** ^**u**^
Urinalysis				
Nitrite (positive)	15 (14.9)	14 (27.5)	1 (2)	**< 0.001** ^**c**^
Pyuria (positive)	60 (59.4)	51 (100)	9 (18)	**< 0.001** ^**c**^
Hematuria (positive)	0 (0)	0 (0)	0 (0)	
Bacteriuria (positive)	58 (57.4)	49 (96.1)	9 (18)	**< 0.001** ^**c**^
Glucosuria (positive)	62 (61.4)	43 (84.3)	19 (38)	**< 0.001** ^**c**^
Proteinuria (positive)	57 (56.4)	30 (58.8)	27 (54)	0.690 ^c^
Urine culture (positive)	36 (35.6)	28 (54.9)	8 (16)	**< 0.001** ^**c**^
Isolated bacteria				0.302 ^f^
Escherichia coli	30 (83.3)	22 (78.6)	8 (100)	
Escherichia coli + Klebsiella pneumoniae	6 (16.7)	6 (21.4)	0 (0)	
Antibacterial treatment				0.101 ^ff^
Fluoroquinolone	33 (55)	25 (49)	8 (88.9)	
Carbapenem	15 (25)	14 (27.5)	1 (11.1)	
Fosfomycin + Nitrofurantoin	12 (20)	12 (23.5)	0 (0)	
Antibacterial treatment (yes)	60 (59.4)	51 (100.0)	9 (18.0)	**< 0.001** ^**c**^
Hospitalization (yes)	19 (18.8)	18 (35.3)	1 (2)	**< 0.001** ^**c**^
**Duration of hospitalization (day)**	0 (0–7)	0 (0–7)	0 (0–3)	**< 0.001** ^**u**^

^u^ Mann Whitney U Test (Monte Carlo),

^f^ Fisher Exact Test(Monte Carlo), ^ff^ Fisher Freeman Halton Test (Monte Carlo), ^c^ Pearson Chi Square Test (Monte Carlo)

min: Minimum; max: Maximum; BMI: body mass index; FBG: fasting blood glucose

No patients died during the follow-up.

BMI scores and HbA1c were higher, and glucosuria and SGLT-2i treatment were more frequent in those patients with UTI (Table 2).

**Table 2:** Comparison of demographic, clinical and laboratory parameters of the patients treated with or without UTI

	UTI		p
	No (n = 41)	Yes (n = 60)	
	n (%) or median (min-max)	n (%) or median (min-max)	
Sex (female)	23 (56.1)	34 (56.7)	0.558 ^c^
Age (year)	57 (45–67)	55.5 (45–67)	0.469 ^u^
BMI (kg/m^2^)	28 (24–34)	32 (25–41)	**< 0.001** ^**u**^
HbA1c (%)	5.8 (5.6–7.8)	7.7 (5.6–11)	**< 0.001** ^**u**^
FBG (mg/dL)	156 (112–234)	156 (112–234)	0.764 ^u^
Glucosuria (present)	11 (26.8)	51 (85.0)	**< 0.001** ^**c**^
SGLT-2i (+)	0 (0.0)	51 (85.0)	**< 0.001** ^**c**^

^u^ Mann Whitney U test (Monte Carlo),

^c^ Pearson Chi square test (Monte Carlo)

min: Minimum; max: Maximum; BMI: body mass index; FBG: fasting blood glucose

**Fig. 1:**
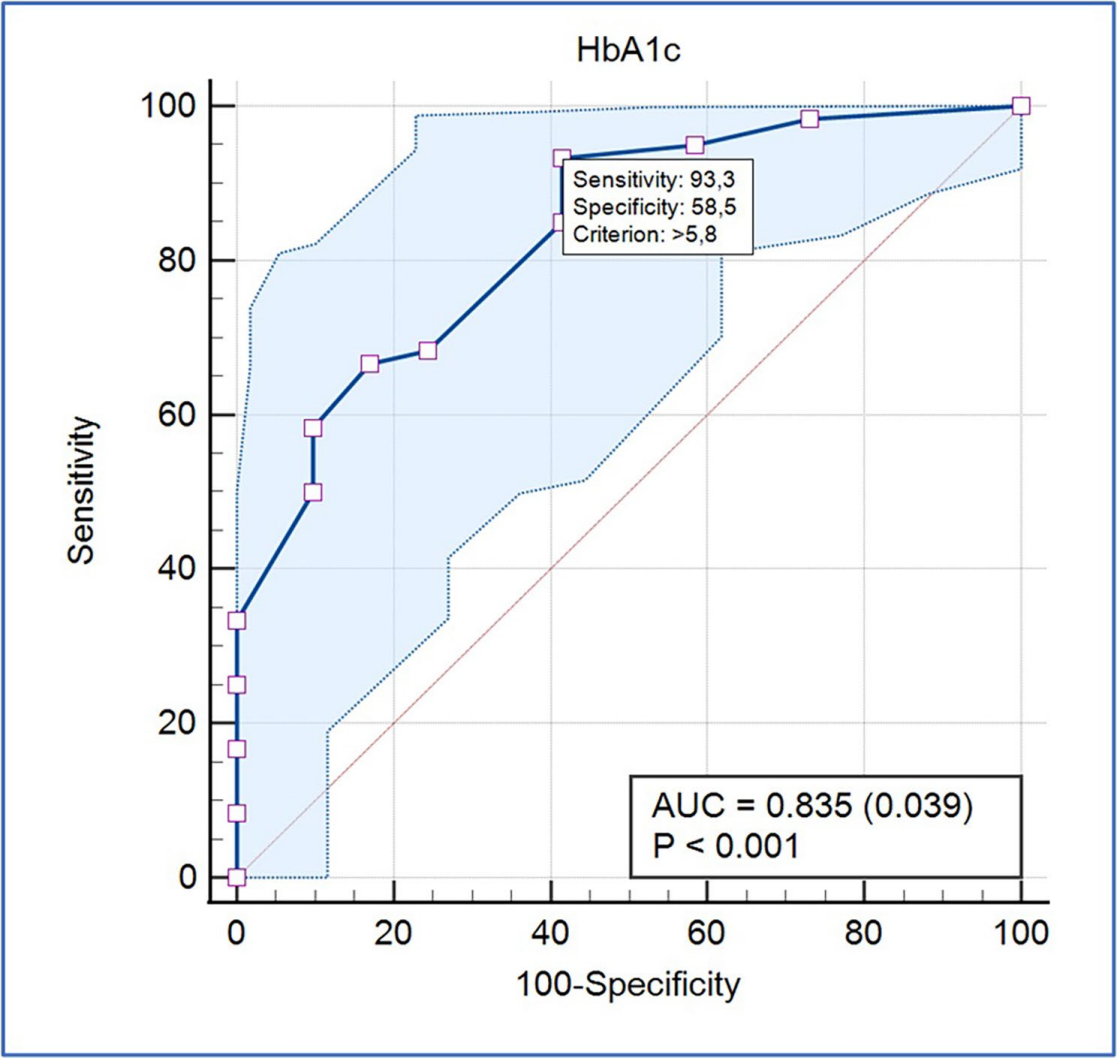
ROC analysis demonstrating the predictive value of HbA1c for UTI.

ROC analysis showed that a HbA1c of > 5.8% was associated with UTI with good accuracy (AUC: 0.835; p < 0.001; sensitivity: 93.3%; and specificity: 58.5%) (Fig. 1).

Multiple logistic regression analysis showed that SGLT-2i use and glucosuria were significant positive predictors for UTI (p = 0.004; Odds Ratio: 1984.013; p = 0.028; and Odds Ratio: 12.480; respectively) (Table 3).

**Table 3:** Multiple logistic regression analysis demonstrating the factors predicting UTI

Dependent Variable: UTI	B	SE.	P	Odds Ratio	95% Confidence Interval for Odds Ratio	
					Lower Bound	Upper Bound
SGLT-2i (+)	-7.593	2.647	**0.004**	1984.013	11.079	355295.550
Age	0.100	0.078	0.198	1.105	0.949	1.287
Sex (female)	-0.254	1.146	0.824	0.775	0.082	7.324
BMI	0.222	0.130	0.089	1.248	0.967	1.612
Glucosuria (present)	-2.524	1.151	**0.028**	12.480	1.307	119.155
FBG	-0.040	0.022	0.074	0.961	0.920	1.004
HbA1c (Categorized by threshold value of 5.8)	1.190	0.984	0.226	3.288	0.478	22.621
Estimated accuracy rates: UTI present (93.3%), UTI absent (95.1%), General (94.1%), P (Model) < 0.001						

B: regression coefficients; SE: Standard error; UTI: Urinary tract infection; BMI: body mass index

## Discussion

Clinical symptoms and laboratory signs of urinary tract infection (UTI) were more frequent in those patients treated with SGLT-2i. Of the patients treated with SGLT-2i, urine culture was positive in more than half, and one third were hospitalized. Besides the association of HbA1c and BMI with UTI, SGLT-2i use and glucosuria were seen to predict UTI.

SGLT-2i use was shown to be associated with UTI in previous studies [1, 3, 26–29]. But the association was not consistently detected, and some other studies did not find any association between SGLT-2 inhibitor use and UTI [4, 5, 8–15]. We concluded that SGLT-2i use predicted the development of UTI. The SGLT-2i class of medications exert their action by inhibiting the reabsorption of glucose in the proximal convoluted tubules by SGLT-2 channels, resulting in glucosuria [30]. Glucosuria has been proposed as a major mechanism facilitating the development of UTI associated with SGLT-2i use [1, 21, 29]. In T2D, the renal threshold for glucose excretion was increased via an over-expression of SGLT-2 channels [31]. SGLT-2i may result in a more enhanced glucosuria response in T2D than expected for a similar glycemic level in a non-diabetic subject. In our study, about 40% of the patients not treated with SGLT-2i did have glucosuria, although the ratio was less than in those treated with SGLT-2i. We showed that glucosuria predicted UTI in those patients with T2D independent of SGLT-2i use, and the predictive value of SGLT-2i for the development of UTI was higher than that of glucosuria. Therefore, not only glucosuria, but also other possible mechanisms seem to play a role in the development of UTI occurring after SGLT-2i use in those patients with T2D. We propose that poor hygiene, inadequate hydration or obesity itself may also contribute to an elevated risk of UTI in those patients with T2D. We showed that there is an association between BMI score and UTI. Besides, obesity may be associated with UTI as a result of associated difficulties in maintaining adequate hygiene standards.

Clinical signs and symptoms suggest UTI was associated with SGLT-2i use in our study. However, some symptoms such as pollakiuria might be a result of glucosuria-induced osmotic diuresis. In one study, pollakiuria due to canagliflozin was found to be associated with natriuresis rather than glucosuria, and shown to be transient [32]. Chronic complications, such as autonomic neuropathy, may be detected following a diagnosis of T2D. In our study, we did not analyze the duration of T2D. We did not perform objective genitourinary autonomic neuropathy tests, which may predispose to UTI or alter the clinical symptoms of UTI. In one study, new-onset nocturia and pollakiuria occurred less frequently after the initiation of SGLT-2i in those patients with T2D and autonomic neuropathy than in those without autonomic neuropathy [33]. Besides, hyperglycemia itself may lead to pollakiuria or nocturia as a result of osmotic diuresis in those patients with significant hyperglycemia. Therefore, rather than the symptoms alone, objective methods such as urinalysis and, more specifically, urine culture are essential to correctly reveal the association of the symptoms with UTI. We included over a hundred patients with T2D for whom a urine culture was performed.

A DURATION-8 study showed an increased risk of UTI after dapagliflozin use, and similar findings were also reported for empagliflozin [1, 3, 6, 26, 34]. Studies comparing different preparations of the SGLT-2i class in terms of UTI are scant. In a meta-analysis, the risk of UTI was found to be similar in different subgroups of the SGLT-2 inhibitor class, and was independent of the dose of SGLT-2i preparation with the exception of dapagliflozin [16]. In that study, high-dose dapagliflozin was associated with an increased risk of UTI. Another systemic meta-analysis suggested that empagliflozin was more likely associated with UTI than dapagliflozin [1]. We included the patients receiving empagliflozin or dapagliflozin, analyzed both together, but could not analyze the other preparations of the SGLT-2 inhibitor class. We treated the patients with the same dose of dapagliflozin or empagliflozin during the follow-up, and could not analyze the effect of the dose of SGLT-2i on the development of UTI.

SGLT-2i use was associated with not only uncomplicated UTI, but also with urosepsis and pyelonephritis in some reports (18–20). However, severe infection associated with SGLT-2i use is less likely, as shown in a previous study where there was a low incidence of severe UTI of 0.4% after SGLT-2i use [17–20]. In our study, one third of the patients receiving SGLT-2i were hospitalized due to the severity of the infection, and SGLT-2i use was associated with the need for hospitalization. We hospitalized the unstable patients, those requiring intravenous antibacterial treatment, or complicated UTI. Neither Pyelonephritis nor urosepsis was not detected in any patients. We also showed that a higher duration of hospitalization was associated with SGLT-2i use. In a meta-analysis, the risk of UTI was similarly greater in different subgroups of the SGLT-2i class, but the risk of upper urinary tract infection increased only where dapagliflozin 10 mg was used [29].

Klebsiella pneumoniae may be isolated from urine culture in those subjects with uncomplicated UTI, where the frequency is 1–2% (35). Klebsiella pneumoniae or Escherichia coli species may be resistant to antibacterial treatment, which may contribute to the need for hospitalization [35]. In our study, although the ratio of bacteria species isolated from the urine culture was similar to that in those patients treated with SGLT-2i or not, Klebsiella pneumoniae was isolated only in those patients treated with SGLT-2i. The frequency of acquired resistance is known to be > 30% for nitrofurantoin, and 10–20% for fluoroquinolones in Klebsiella pneumoniae [35]. About 25% of Klebsiella pneumoniae species produce extended-spectrum beta-lactamase [35]. Due to the reduced effect of fosfomycin or nitrofurantoin, oral treatment is more challenging for infection with ESBL-positive Klebsiella pneumoniae strains than ESBL-positive Escherichia coli strains [35]. Isolation of the Klebsiella pneumoniae strain in those patients treated with SGLT-2i might affect the need for hospitalization or the administration route of antibacterial treatment. Therefore, a urine culture study is so important in those patients with T2D, especially in those treated with SGLT-2i.

SGLT-2 inhibitors were also shown to be associated with an increased risk of genital mycotic infections especially in those patients with a history of genital mycotic infection and in uncircumcised males [3–5]. Genital infections may be co-existent with UTI in patients with T2D. We analyzed those patients with UTIs alone, and excluded those with any signs or symptoms of genital infections.

In our study, BMI score, FBG and HbA1c levels were higher in those patients treated with SGLT-2i than in those not treated with SGLT-2i. Actually, due to the known efficacy of SGLT-2 inhibitors on glycemic control in T2D, and the beneficial effects on body weight, we prescribed SGLT-2 inhibitors especially in those patients with a higher HbA1c or higher BMI score [6, 31]. Our findings suggested that a higher BMI score or HbA1c was associated with UTI. Among those patients in our study, who were treated with antidiabetic medications, the HbA1c cut-off for association with UTI was as low as 5.8%. According to our study, the predictive role of SGLT-2i use for UTI seems to be independent of BMI score or HbA1c.

### Strength and limitations

We included an important number of patients with T2D for whom a urine culture was performed. We did not analyze the members of the SGLT-2i class other than empagliflozin or dapagliflozin. We analyzed those patients treated with empagliflozin or dapagliflozin together. We excluded patients with any signs or symptoms of genital infections, but could not analyze the presence of genitourinary autonomic dysfunction. We could not analyze the duration of T2D, or the duration between the emergence of UTI and the initiation of SGLT-2i.

## Conclusion

Studies analyzing urine cultures in the investigation of the association of SGLT-2i use with UTI in T2D are limited. We found that SGLT-2i use and glucosuria predicted UTI in those patients with T2D. Our findings suggest that performing a urine culture is very important in choosing the type and administration route of antibacterial treatment, especially in those patients receiving SGLT-2i treatment. It seems that glucosuria is not the only mechanism underlying the development of UTI in patients treated with SGLT-2i. The effect of SGLT-2i on the development of UTI is independent of the baseline BMI score or HbA1c. Future studies, which include a large sample of subjects with T2D receiving SGLT-2i treatment, may indicate the other possible factors contributing to UTI.

## Data Availability

The datasets generated during and/or analyzed during the current study are available from the corresponding author on reasonable request. The authors assure that this paper has not been published before nor has been submitted for publication to another scientific journal.
